# Standardizing pCR/MPR Assessment in NSCLC After Neoadjuvant Therapy: Challenges and Perspectives

**DOI:** 10.3390/cancers18162676

**Published:** 2026-08-18

**Authors:** Andrea Ascione, Flavia Adotti, Luigi Vittori, Caterina Chiappetta, Paolo Graziano

**Affiliations:** 1 Department of Experimental Medicine, Sapienza University of Rome, 00161 Rome, Italy; andrea.ascione@uniroma1.it (A.A.);; 2 Pathology Unit of Policlinico Umberto I, Sapienza University of Rome, 00161 Rome, Italy; 3 Department of Radiological, Oncological and Pathological Sciences, Sapienza University of Rome, 00161 Rome, Italy

**Keywords:** non-small cell lung cancer, neoadjuvant therapy, major pathologic response, pathologic complete response, residual viable tumor, IASLC, molecular residual disease, circulating tumor DNA, digital pathology

## Abstract

The expansion of neoadjuvant immune checkpoint blockade, chemoimmunotherapy, and targeted therapies in oncogene-driven tumors is reshaping the management of resectable non-small cell lung cancer (NSCLC). Major pathologic response (MPR) and pathologic complete response (pCR), defined respectively as 10% or less residual viable tumor (RVT) within the primary tumor bed and the complete absence of viable tumor in both the resected primary tumor and sampled regional lymph nodes, have become widely adopted early efficacy endpoints. Both are consistently associated with favorable survival outcomes at the patient level, although their validity as trial-level surrogates for long-term outcomes remains incompletely established. Accurate pathologic response assessment requires rigorous standardization of gross specimen handling, tumor bed sampling, and microscopic quantification of viable tumor, necrosis, and stroma. International recommendations have improved methodological harmonization, and reproducibility studies have demonstrated good interobserver agreement when standardized protocols are applied. Increasing evidence also indicates that RVT behaves as a continuous prognostic variable and that integration of primary-tumor and nodal response may improve postoperative risk stratification. Beyond quantification of RVT alone, additional histologic characteristics of the residual tumor, together with stromal and immune therapy-related features, may provide complementary prognostic information. Digital pathology and artificial intelligence may support more reproducible quantitative assessment, whereas circulating tumor DNA-based molecular residual disease evaluation may capture systemic risk not represented by the resection specimen. These emerging approaches remain investigational and require prospective validation. This review critically examines the methodological standardization, biological interpretation, and prognostic validation of pathologic response in resectable NSCLC, and discusses future strategies integrating histopathologic, digital, and molecular variables into post-neoadjuvant risk assessment.

## Simple Summary

Treatment given before surgery is increasingly used for patients with resectable non-small cell lung cancer. After surgery, pathologists examine the removed tumor to determine how much living cancer remains and how the surrounding tissue has changed. This assessment can provide early information on treatment effectiveness and on the patient’s risk of recurrence, but differences in specimen handling, sampling, and interpretation may affect its reliability. This review explains how pathologic response should be assessed, how it relates to long-term outcomes, and how findings in the primary tumor and lymph nodes can be combined. It also discusses emerging tools, including digital pathology, artificial intelligence, and blood-based detection of residual cancer. A more standardized and integrated approach could improve comparison between clinical trials, strengthen postoperative risk assessment, and support the future development of more personalized treatment strategies.

## 1. Introduction

Lung cancer remains the leading cause of cancer-related mortality worldwide, with approximately 80–85% of these cases being classified as non-small cell lung cancer (NSCLC) [1,2]. Despite progress in diagnostic and therapeutic strategies, only a minority of patients present with disease amenable to curative-intent resection at diagnosis, making accurate staging and optimal perioperative management critical determinants of long-term outcome [1].

In this setting, neoadjuvant therapy has progressively become an integral component of the management of potentially resectable NSCLC, evolving from a secondary option to a standard element of multimodal treatment strategies [3,4]. Early meta-analyses showed that preoperative chemotherapy improved survival compared with surgery alone, while offering the theoretical advantages of earlier systemic treatment, improved treatment compliance, and in vivo assessment of therapeutic sensitivity [5,6]. Initially introduced in the era of chemotherapy, its role has substantially expanded with the incorporation of immune checkpoint inhibitors (ICIs) and, more recently, the investigation of targeted therapies in oncogene-driven tumors [4,7,8].

With the broader implementation of neoadjuvant strategies, treatment evaluation has increasingly shifted beyond radiologic and long-term survival endpoints. Tissue-based measures of therapeutic effect now play a central role, as histopathology can directly quantify residual viable tumor (RVT) and characterize treatment-related stromal and immune modifications [3,9].

While pathologic complete response (pCR) had long been recognized as an oncologic endpoint, the introduction of major pathologic response (MPR) represented a major step toward standardization in NSCLC [4,8]. Compared with pCR, MPR is observed more frequently and has shown meaningful associations with long-term outcomes at the patient level, thereby facilitating the clinical development of neoadjuvant strategies [10,11,12].

However, the apparent simplicity of RVT percentage conceals a more complex biological reality that is only partially captured by the concepts of MPR and pCR. Tumor response after chemotherapy, immunotherapy, or targeted therapy reflects distinct interactions among tumor cell death, stromal remodeling, host immune activation, and therapy-specific biological effects, and may generate distinct patterns of regression [13,14].

In addition, RVT percentage alone may not fully capture other biologically relevant dimensions of response, including immune contexture, intratumor heterogeneity, nodal regression patterns, and molecular residual disease (MRD) detectable through circulating tumor DNA (ctDNA) assays [15,16,17].

At the same time, the increasing use of MPR and pCR as early endpoints in clinical trials has raised substantial methodological and interpretative issues. Although strong associations with survival outcomes have been demonstrated at the patient level, their validity as surrogate endpoints at the trial level remains incompletely established [12,17,18].

Taken together, these observations indicate that pathologic response should not be considered merely a descriptive parameter, but rather a multidimensional readout of therapeutic efficacy with implications for clinical decision-making, trial design, and biomarker development. There is therefore a need to critically reassess both the methodological basis and the biological interpretation of pathologic response in the neoadjuvant setting.

In this review, we examine the historical evolution, methodological standardization, and biological foundations of pathologic response assessment in NSCLC and discuss emerging strategies integrating morphologic, immune, and molecular dimensions into a more comprehensive framework of treatment evaluation.

### Review Methodology

This article is intended as a narrative critical review rather than a systematic review. Relevant literature was identified through targeted searches of PubMed/MEDLINE and by screening the reference lists of key articles. Searches were last updated in August 2026 and used combinations of terms including “non-small cell lung cancer”, “neoadjuvant therapy”, “pathologic response”, “major pathologic response”, “pathologic complete response”, “residual viable tumor”, “tumor bed”, “nodal response”, “ctDNA”, “molecular residual disease”, “digital pathology”, “artificial intelligence”, “targeted therapy”, “EGFR”, “ALK”, and “tyrosine kinase inhibitor”. Priority was given to IASLC recommendations, randomized and prospective neoadjuvant trials, meta-analyses, reproducibility studies, and pathology-focused reviews addressing relevant topics. English-language articles were prioritized. Case reports and small series were considered only when they addressed underrepresented settings, particularly targeted therapy-treated tumors. Because the aim was critical synthesis rather than quantitative evidence aggregation, formal risk-of-bias assessment and meta-analytic methods were not applied.

## 2. Historical Emergence of Pathologic Response as a Readout of Treatment Effect

The evolution of pathologic response as a widely accepted endpoint in NSCLC trials reflects both the growth of perioperative strategies and the limitations of relying solely on radiologic findings or long-term survival. As neoadjuvant treatment became standard, pathologic response offered a necessary, earlier, and more biologically informative measure of efficacy for trial design and translational research.

### 2.1. Early Chemotherapy Experience

The concept of pathologic response in NSCLC was initially developed in the setting of induction chemotherapy. Early studies showed that the amount of residual viable tumor (RVT) in resection specimens after preoperative treatment was associated with postoperative outcomes, suggesting that histopathologic regression could serve as an early readout of treatment effect [6,11,19].

These observations culminated in the formalization of the concept of MPR, generally defined as 10% or less residual viable tumor within the primary tumor bed. Compared with pCR, which remained relatively infrequent after chemotherapy alone, MPR offered a more common and quantifiable endpoint, thereby increasing its practical utility for clinical studies and cross-trial comparisons [10,11].

The chemotherapy era also highlighted important methodological limitations in the form of heterogeneous approaches to specimen sampling, tumor bed identification, and estimation of RVT, severely limiting reproducibility across study cohorts [11,19]. In addition, nodal response was inconsistently incorporated into pathologic scoring systems, and no universally accepted reporting framework was available during this phase of development [3,11]. Thus, although pathologic regression had already demonstrated potential clinical relevance, its implementation remained incompletely standardized until later multidisciplinary efforts toward harmonization [3].

Nevertheless, this period established a key conceptual principle that continues to shape the field today: the degree of RVT after neoadjuvant therapy contains prognostic information complementary to the postoperative pathologic stage.

### 2.2. Immunotherapy Era

The introduction of ICIs in the neoadjuvant setting represented a major turning point in both treatment efficacy and pathologic interpretation. Early prospective studies of programmed cell death protein 1 (PD-1) blockade demonstrated unexpectedly high rates of MPR despite modest or inconsistent radiologic shrinkage, emphasizing the limitations of imaging-based response criteria in this context [9,20].

Subsequent randomized trials combining ICIs with platinum-based chemotherapy demonstrated substantial increases in both MPR and pCR compared with chemotherapy alone, establishing pathologic response as a prominent early efficacy endpoint in perioperative clinical trials [4,8]. In this setting, pathologic response became not only a descriptive histologic measure, but also a regulatory and strategic endpoint capable of accelerating therapeutic evaluation.

At the same time, immunotherapy introduced novel regression patterns, substantially different from those historically observed after chemotherapy [13,14]. Such findings required pathologists to distinguish true residual malignancy from therapy-induced regression and inflammatory mimicry, exposing once again the need for rigorous standardization of specimen handling and scoring systems. This ultimately led to multidisciplinary recommendations for pathologic assessment after neoadjuvant therapy, including formal guidance on tumor bed identification, sampling strategies, and quantification of RVT [3].

In parallel, pooled analyses and secondary studies suggested that RVT percentage behaves as a continuous prognostic variable rather than a purely dichotomous threshold [12,17,18].

### 2.3. Targeted Therapy in Oncogene-Driven Tumors

The recent implementation of neoadjuvant strategies to molecularly selected NSCLC has introduced a new phase in the evolution of pathologic response assessment. In tumors harboring actionable genomic alterations—most notably epidermal growth factor receptor (EGFR) mutations and anaplastic lymphoma kinase (ALK) rearrangements—targeted therapies are increasingly being explored as perioperative alternatives to immunotherapy-based approaches, particularly due to the lower sensitivity of these tumors to ICIs [21,22].

Evidence is most developed in EGFR-mutated disease. The EMERGING-CTONG1103 trial provided early evidence of activity with neoadjuvant erlotinib, while the phase III NeoADAURA trial subsequently demonstrated significantly higher MPR rates with neoadjuvant osimertinib, administered alone or with chemotherapy, than with chemotherapy alone [22]. In ALK-rearranged NSCLC, the phase II ALNEO study reported encouraging pathologic responses and supported the surgical feasibility of neoadjuvant alectinib [23].

The biological effects of targeted therapy differ from those induced by chemotherapy or immunotherapy. Tyrosine kinase inhibitors (TKIs) primarily suppress oncogenic signaling and tumor-cell proliferation and may therefore generate regression beds whose morphologic and biological characteristics differ from those produced by direct cytotoxicity or immune-mediated clearance. Consequently, the prognostic meaning of a given RVT percentage—and the validity of conventional MPR and pCR thresholds—cannot be assumed to be identical across therapeutic classes.

Long-term data linking pathologic response after neoadjuvant targeted therapy to survival remain limited. Future studies should determine whether morphology-based assessment can be strengthened by integration with MRD and genomic biomarkers. More broadly, the expansion of pathologic response assessment across therapeutic classes has made harmonized definitions, sampling protocols, and scoring methods increasingly essential.

## 3. Standardization of Pathologic Assessment: From Variability to Harmonization

The International Association for the Study of Lung Cancer (IASLC) multidisciplinary recommendations represented the first comprehensive lung-specific effort to harmonize post-neoadjuvant pathologic assessment. They established a common framework in which the tumor bed is evaluated through viable tumor, necrosis, and stroma, with these components summing to 100% [3]. This standardization is relevant not only to clinical trials but also to routine practice, particularly as pCR and MPR are increasingly incorporated into postoperative risk assessment and multidisciplinary discussion [24,25,26].

### 3.1. Tumor Bed Identification

The tumor bed corresponds to the anatomic area previously occupied by the pretreatment tumor, regardless of whether a grossly evident residual mass remains at resection. In cases with limited response, it may still appear as a residual mass or pleural puckering [3]. Conversely, in cases approaching MPR or pCR, the lesion may be reduced to a subtle scar or an ill-defined fibrotic zone. Correlation with pretreatment and preoperative imaging is therefore essential, particularly when the lesion is poorly defined macroscopically [27].

Imaging should be regarded as an integral complement to gross examination, but radiologic size reduction should not be equated with histopathologic regression. Radiologic and pathologic responses may be markedly discordant, and conventional RECIST criteria may underestimate treatment efficacy when persistent radiographic abnormalities largely reflect fibrosis, inflammation, or other components of the regression bed rather than viable tumor [20,28]. Several caveats complicate tumor bed recognition. Cavitation may distort the original lesion and obscure the relationship between necrosis and residual tumor [25,27]. Dense fibrosis, fibroelastotic scarring, treatment-related inflammatory remodeling and organizing pneumonia may grossly mimic residual carcinoma or blur the boundary between true tumor bed and adjacent reactive lung [3,24,25]. Microscopically, reactive pneumocyte atypia, histiocytic infiltrates, cholesterol clefts and organizing inflammatory exudates may further complicate interpretation [29]. Immunohistochemistry may be useful in selected difficult cases, particularly when small residual epithelial clusters must be distinguished from macrophage-rich inflammation or reactive pneumocytes. Broad-spectrum cytokeratins and histiocytic markers may support this distinction, while lineage markers can be added when confirmation of tumor phenotype is required. However, ancillary stains should not be routinely used for response scoring and cannot replace morphologic assessment [25,29]. Because of these pitfalls, complex cases require large peripheral sampling and rigorous radiologic-pathologic correlation [3,24]. This problem is not entirely treatment-specific: regression-like histologic appearances have also been described in untreated carcinomas, underscoring that regressive morphology must be interpreted in proper clinico-radiologic context [30].

### 3.2. Gross Sampling Strategies

Accurate gross sampling is the first essential step in pathologic response assessment, because microscopic quantification depends on adequate representation of the tumor bed [3,25]. In the IASLC recommendations, tumor beds measuring 3 cm or less should be submitted entirely. For larger lesions, the specimen should be sliced along the longest tumor bed axis; one mapped 0.5 cm thick section should be submitted completely, with additional sections obtained per centimeter of tumor bed and peripheral sections including adjacent uninvolved lung to define the tumor bed boundaries [3,27]. If initial microscopic review suggests pCR, the remaining tumor bed should be entirely embedded [3,25]. This approach represents the current international reference standard and should be distinguished from alternative or more extensive sampling strategies proposed in individual studies.

Several practical factors may justify more extensive sampling than the minimum recommended approach. Correct tumor bed recognition, representative block selection, and awareness of treatment-related mimics can each influence final response classification. This is particularly relevant because post-treatment NSCLC regression may be spatially heterogeneous, with small discontinuous foci of viable carcinoma dispersed within fibrosis, necrosis, and inflammation [24,31]. Limited sampling may therefore lead to false pCR classification or underestimation of RVT, especially in cases with marked regression.

Alternative strategies have been proposed to reduce this risk, but they should be regarded as practice-oriented refinements rather than universally adopted standards. Saqi et al. suggested complete submission of the visible tumor bed in cases of suspected pCR, complete embedding for tumors 3 cm or smaller, and at least 50% sampling of larger tumor beds, while also linking gross representativeness to weighted calculations of RVT [32]. Berezowska et al. noted that some groups advocate embedding every second slice in tumors larger than 3 cm, resulting in microscopic evaluation of at least 50% of the tumor bed [24]. Simulation data further suggest that the traditional rule of one block per centimeter may be insufficient in a subset of cases, with accurate classification requiring complete embedding of smaller tumors and, for larger tumor beds, up to approximately 21 sections to achieve high accuracy [33,34].

Thus, no fixed “magic number” of sections can be universally applied. Sampling should follow the IASLC framework as the minimum standardized reference, while being adapted to tumor size, gross definition, suspected degree of regression, and the need to exclude RVT in cases approaching pCR. Irrespective of the specific protocol adopted, mathematical refinements in response calculation cannot compensate for inadequate tissue representation. Correct identification and comprehensive mapping of the tumor bed remain the foundation of any subsequent microscopic score.

### 3.3. Microscopic Quantification

Microscopic response assessment is based on estimating the proportions of viable neoplastic cells, necrosis, and stroma within the tumor bed. In the IASLC framework, these components are generally scored in 10% increments, with finer single-digit estimates reserved for very low amounts of RVT near clinically relevant cutoffs [3,24,27]. This approach provides a pragmatic common language for routine reporting and multicenter clinical trials. It has been adopted across studies of chemotherapy and chemoimmunotherapy and currently represents the principal framework used when evaluating newer neoadjuvant strategies [25].

Earlier outcome studies established pathologic response as a strong histologic correlate of survival after neoadjuvant chemotherapy, providing the basis for RVT-based response thresholds [10,35,36]. In contemporary clinical trials, MPR is defined as 10% or less RVT within the primary tumor bed, whereas pCR requires complete absence of viable tumor in both the resected primary tumor and all sampled regional lymph nodes [3,4].

However, histotype-specific analyses suggest that the biological and prognostic meaning of a given RVT percentage may not be identical across NSCLC subtypes. Squamous cell carcinomas tend to have a lower median RVT and higher MPR rates than adenocarcinomas, and different survival-associated thresholds have been reported in chemotherapy-treated cohorts [37]. These observations do not invalidate the operational value of the 10% cutoff, which remains the most widely accepted and reproducible threshold, but suggest that its interpretation may be partly histology-dependent [3,38].

Necrosis should not be interpreted as an independent surrogate of response, because it may represent pretreatment tumor biology, treatment-induced change, or both. Therefore, it lacks sufficient specificity as a stand-alone regression metric. In the CheckMate 816 pathologic analysis, RVT and regression were more informative than necrosis with respect to event-free survival (EFS), whereas necrosis was similar between treatment arms and did not parallel treatment benefit in a clinically useful way [17].

In neoadjuvant chemoimmunotherapy, additional methodological differences may also arise from whether response is assessed in the primary tumor alone or in combination with nodal disease; in this setting, IASLC-based primary-tumor assessment and combined primary-tumor-plus-lymph-node criteria do not always classify the same patients as MPR, underscoring the need to preserve a common standard when reporting pathologic response [39].

### 3.4. Interobserver Reproducibility

Interobserver reproducibility is central to the reliability of MPR and pCR as clinical and trial endpoints [3,24]. The IASLC multicenter reproducibility study showed good agreement in classifying specimens according to the 10% MPR cutoff, with the greatest concordance at the extremes of response, namely absence of RVT or extensive residual disease [29]. These findings suggest that variability depends less on the conceptual validity of the cutoff itself than on the practical challenge of identifying the same tumor bed and interpreting treatment-related changes consistently.

In the earlier single-center study by Weissferdt et al., MPR remained significantly associated with overall survival (OS) when assessed by either an experienced or a specifically trained observer, supporting the robustness of RVT-based scoring [40].

A Korean interobserver study further refined this issue. Despite high overall reproducibility in terms of tumor bed annotation and MPR classification, agreement decreased when the area of tumor bed was limited or poorly defined due to therapy-associated or reactive changes. This problem appeared particularly relevant in squamous cell carcinoma, where central location, bronchovascular fibrosis, airway-centered scarring, and post-obstructive change may complicate tumor bed delineation more than in peripheral adenocarcinoma [38]. Additional problematic scenarios included mucinous tumors, reactive pneumocytes versus residual lepidic growth, and cases with numerous minute foci of viable tumor dispersed within abundant stroma [24,29,38].

### 3.5. Weighted Versus Unweighted Models

Various summary measures, including weighted and unweighted models, have been used to quantify RVT across histologic slides. In the unweighted approach, each slide contributes equally to the final estimate; in weighted models, each slide is adjusted according to the proportion of total tumor bed area it represents. This approach is methodologically attractive, and the MPR calculator proposed by Saqi and colleagues was developed to formalize this area-adjusted computation and to standardize computation of RVT, necrosis and stroma [32]. More broadly, interest in alternative quantitative models reflects a longstanding effort to capture the spatial extent of residual disease more faithfully. For example, Yamane et al. proposed the area of residual tumor as a prognostically relevant parameter after neoadjuvant therapy, arguing that conventional size-based or purely proportional estimates may not fully reflect residual tumor burden in irregular regression beds [41].

Available evidence, however, suggests that weighting has limited impact on MPR classification. In the IASLC reproducibility study and in the Korean interobserver study, weighted and unweighted methods yielded similar results, indicating that differences in scoring model contributed less to variability than differences in case interpretation [29,38].

### 3.6. Standardized Reporting and Communication in the Tumor Board

A standardized pathology report is the natural endpoint of harmonized gross and microscopic assessment. It should document the neoadjuvant regimen, identify the assessed primary tumor bed, report the estimated percentages of RVT, necrosis, and stroma summing to 100%, state whether MPR or pCR has been achieved, and integrate these findings with ypTNM stage [3,27]. Since pCR in lung cancer requires the absence of viable tumor in both the primary tumor and sampled lymph nodes, nodal response must not be considered optional, representing instead an integral part of the process [3,4].

Standardization also requires bidirectional communication within the multidisciplinary team. Surgeons and oncologists should provide treatment history, preoperative imaging, operative findings, and nodal sampling information, because post-treatment interpretation is inseparable from clinico-radiologic context. Conversely, the pathologist should clearly communicate whether the response category refers to the primary tumor alone or incorporates nodal disease, whether primary and nodal responses are concordant, and whether the final categorization is limited by sampling or interpretive uncertainty [3,25,27]. This is particularly important after neoadjuvant chemoimmunotherapy, where discordant responses between the primary tumor and lymph nodes are well documented and may influence prognostic interpretation [39,42]. Accordingly, reports should make explicit whether MPR refers to the IASLC primary-tumor-based assessment or to a combined primary-tumor-plus-nodal framework, rather than assuming that these approaches are interchangeable [3,39].

From a practical perspective, the value of standardized pathologic response assessment lies not only in reproducible slide-based scoring, but also in its ability to function as a common language linking pathology, radiology, surgery, and medical oncology in post-neoadjuvant decision-making [3,17]. Histopathology should remain the backbone of this assessment, but the next phase of harmonization will likely involve structured integration of validated adjunct biomarkers rather than morphology alone.

To avoid ambiguity in reporting and multidisciplinary communication, pathologic response endpoints should be explicitly linked to the anatomic compartment assessed and to their current level of validation (Box 1). Table 1 summarizes the main response categories and clarifies which are established standards and which remain investigational or descriptive.

Box 1 **Practical reporting checklist for multidisciplinary communication.**

In post-neoadjuvant NSCLC specimens, the pathology report should explicitly state: (i) the neoadjuvant regimen received; (ii) the size and location of the assessed primary tumor bed; (iii) whether the tumor bed was entirely submitted or, if not, the approximate extent of sampling; (iv) the percentages of RVT, necrosis, and stroma/inflammation in the primary tumor bed, summing to 100%; (v) whether primary-tumor MPR has been achieved; (vi) whether pCR has been achieved, defined by absence of viable tumor in both primary tumor and sampled lymph nodes; (vii) the number and stations of examined lymph nodes, the presence or absence of residual nodal metastasis, and any evident nodal treatment effect; (viii) the final ypTNM stage; and (ix) relevant biomarkers, including PD-L1 expression and actionable genomic alterations when available. When primary-tumor and nodal responses are discordant, this should be highlighted explicitly rather than collapsed into a single ambiguous response category.

## 4. Biological Interpretation of Tumor Regression

Increasing evidence indicates that qualitative features of the tumor bed, including stromal remodeling and immune contexture, reflect distinct biological features and may provide additional prognostic and predictive information [43]. As already mentioned, the tumor bed should be conceptualized as a composite of viable tumor, necrosis, and stroma, all contributing to 100% of the evaluated area [3]. Within this framework, understanding how tumors regress—rather than solely how much remains—becomes essential.

### 4.1. Chemotherapy-Induced Patterns

Cytotoxic chemotherapy induces tumor regression primarily through direct DNA damage. Therefore, apoptotic or necrotic cell death, histologically resulting in coagulative necrosis, hypocellularity and stromal fibrosis, may occupy a substantial proportion of the tumor bed [43]. Necrosis may appear as confluent or patchy areas with cellular debris and ghost outlines, while fibrosis reflects reparative processes with collagen deposition and fibroblast proliferation. The stromal compartment also includes heterogeneous inflammatory infiltrates, lacking the structured architecture seen in immune-mediated responses.

Lunardi et al. highlighted the prognostic relevance of stromal components in neoadjuvant chemotherapy settings [44]. Morphometric analyses suggest that MPR is frequently accompanied by extensive fibrosis, while greater fibrotic remodeling appears to be associated with improved disease-free survival (DFS). Notably, the prognostic impact of inflammation seems to depend more on the immune contexture and cellular composition of the infiltrate than on its percentage alone [44].

These observations suggest that the stromal compartment may contain information not entirely captured by RVT percentage. However, fibrosis and necrosis are neither treatment-specific nor reliable independent measures of tumor eradication, because viable tumor may persist within fibrotic or necrotic areas and similar changes may also spontaneously occur in untreated tumors.

### 4.2. Immunotherapy-Related Regression Bed

ICIs and ICI-based combinations induce patterns of tumor regression characterized by immune-mediated tumor clearance and complex stromal remodeling. These changes have led to the conceptualization of the immunotherapy-related regression bed, defined as the original tumor site replaced by fibroinflammatory tissue following immune-mediated tumor cell eradication [43]. Importantly, this regression bed accounts for the well-documented discrepancy between radiologic tumor size and the actual RVT burden [14].

**Figure 1:**
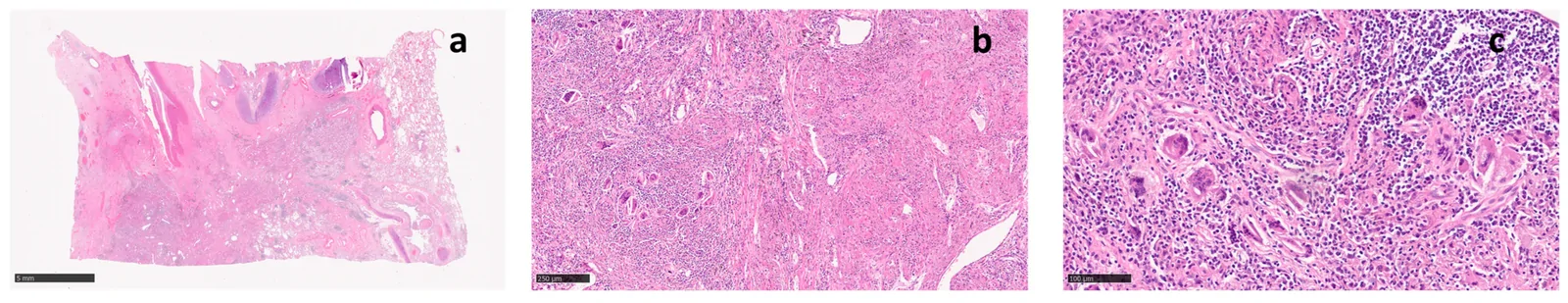
Tumor bed in a lung resection specimen after neoadjuvant chemoimmunotherapy. Low-power ((**a**) hematoxylin and eosin; original magnification 0.5×), medium-power ((**b**) hematoxylin and eosin; original magnification 7.5×), and high-power views ((**c**) hematoxylin and eosin; original magnification 20×) show pCR characterized by extensive fibrosis and a multinucleated giant-cell reaction, without residual viable neoplastic cells.

Unlike the predominantly cytotoxic effects associated with chemotherapy, immunotherapy may generate an immune-rich regression bed in which fibrosis, inflammation, tumor-cell death, and tissue repair coexist (Figure 1).

However, because many contemporary specimens derive from combined chemoimmunotherapy rather than ICI monotherapy, individual morphologic findings cannot always be attributed exclusively to immune-mediated or chemotherapy-mediated effects. Overlapping regression patterns are therefore expected in this setting.

The changes described may obscure small residual tumor foci or, conversely, mimic persistent malignancy [43]. In this context, Cottrell et al. [14] provided a pivotal conceptual framework by defining immune-related pathologic response (irPR) as the result of three coordinated processes: (I) immune activation, evidenced by dense tumor-infiltrating lymphocytes, histiocytes and tertiary lymphoid structures; (II) tumor cell death; (III) tissue repair phenomena such as neovascularization and proliferative fibrosis. These features are typically more prominent in tumors achieving MPR compared with non-responders, as originally described following anti-PD-1 therapy [45]. These criteria exhibited a good degree of interobserver reproducibility when assessed by different pathologists [14].

The spatial distribution of response may also differ from that observed after chemotherapy. An “outside-in” pattern has been described, with regression frequently developing at the periphery of residual tumor deposits, with practical implications for tumor-bed delineation, sampling, and RVT quantification [14]. Immune activation may be diffuse or spatially organized and can include T lymphocytes, B cells, plasma cells, macrophages, foamy histiocytes, cholesterol clefts, granulomatous reaction, and lipid-rich necrosis [43,45]. Although these findings support an immune-mediated treatment effect, none is individually specific, and they should not replace direct identification and quantification of viable tumor.

Among immune-related features, tertiary lymphoid structures are of particular biological interest. Their abundance and maturation have been associated with MPR and improved DFS in selected neoadjuvant chemoimmunotherapy studies, suggesting that organized immune activation may carry biological and prognostic information beyond diffuse inflammation alone [46]. TLS may also reflect coordinated T- and B-cell activation and sustained local antigen presentation, although their role in routine response assessment remains investigational [45,47]. Tissue repair, including proliferative fibrosis and neovascularization, is another component of the immunotherapy-related regression bed; in chemoimmunotherapy-treated specimens, these reparative features may coexist with cytotoxic changes and should be interpreted in the context of the administered regimen [14,45].

Overall, immune-related regression criteria provide a biologically informative description of treatment effect, but current standardized assessment continues to rely primarily on RVT within the entire tumor bed. Qualitative immune features should therefore be regarded as complementary descriptors rather than established alternative response endpoints.

### 4.3. Targeted Therapy-Induced Regression Patterns

Targeted therapies, particularly TKIs directed against oncogenic drivers such as EGFR and ALK, may induce regression patterns that differ from those observed after chemotherapy or immunotherapy. These differences may reflect their predominant mechanism of action, which involves suppression of oncogenic signaling and inhibition of tumor-cell proliferation, although cytotoxic effects may also occur [24,48].

**Figure 2:**
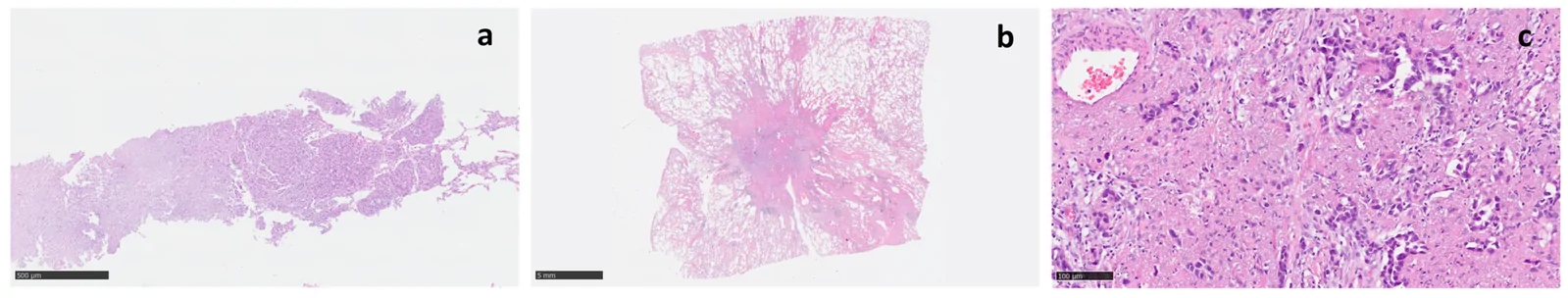
Lung adenocarcinoma before and after neoadjuvant tyrosine kinase inhibitor therapy. Percutaneous biopsy of lung adenocarcinoma ((**a**) hematoxylin and eosin; original magnification 5×) and low-power ((**b**) hematoxylin and eosin; original magnification 0.5×) and high-power views ((**c**) hematoxylin and eosin; original magnification 15×) of the corresponding surgical specimen show residual tumor cells within a fibrotic background.

Available pathologic evidence remains limited, and a reproducible, therapy-specific regression pattern for neoadjuvant TKIs has not yet been firmly established. Compared with ICI-treated tumors, and acknowledging that most comparisons are indirect, reported specimens generally show less prominent immune activation, with reduced tumor cellularity accompanied by variable degrees of fibrosis and necrosis and by persistent foci of viable carcinoma (Figure 2) [24,48].

These observations derive from relatively small and heterogeneous clinical series and should therefore be interpreted as descriptive findings rather than validated treatment-specific histologic criteria.

Residual viable tumor cells may persist at low density within a remodeled tumor bed despite marked radiologic or pathologic response [48]. However, morphologically viable cells cannot be assumed to be dormant, non-proliferative, or clinically irrelevant on histologic grounds alone. Conversely, a low RVT percentage does not necessarily indicate complete biological eradication of the tumor or exclude the persistence of potentially resistant subclones. For the same reason, conventional MPR and pCR thresholds should not be automatically assumed to have identical prognostic meaning after targeted therapy without dedicated validation.

Selective pressure exerted by TKIs may favor the persistence or expansion of resistant subclones, but the relationship between molecular resistance and spatial patterns of regression in neoadjuvant resection specimens remains insufficiently defined [48,49].

Nevertheless, the presence and clonal expansion of resistant populations require molecular demonstration and cannot be inferred solely from the morphology or distribution of residual tumor cells. Integration of histopathology with molecular profiling of residual tissue and ctDNA-based MRD assessment will therefore be necessary to determine the biological and prognostic significance of residual disease after neoadjuvant targeted therapy. At present, targeted therapy-treated specimens should therefore be reported using standardized RVT-based criteria, while explicitly acknowledging that the prognostic meaning of these thresholds in TKI-treated tumors remains insufficiently validated.

To emphasize the distinction between descriptive regression features and validated response criteria, Table 2 summarizes the main therapy-related patterns and interpretative caveats across chemotherapy, ICI-based therapy, chemoimmunotherapy, and targeted therapy.

## 5. Prognostic Validation and Conceptual Refinement

The role of pathologic response became central because OS data require prolonged follow-up, while radiologic response often fails to distinguish RVT from treatment-induced regression [25,26]. Pathologic response is therefore increasingly used as an early indicator of treatment efficacy. At the same time, its interpretation requires a clear distinction between prognostic value and formal surrogate validity [12].

### 5.1. MPR as a Robust Patient-Level Marker

The conceptual foundation for MPR as an endpoint in resectable NSCLC was established by Hellmann et al., who proposed that ≤10% RVT was strongly associated with survival, reflected treatment effect, and could serve as a practical early endpoint in clinical trials [10]. Larger meta-analytic datasets have subsequently reinforced this framework.

The most comprehensive recent evidence comes from the meta-analysis by Waser et al., which showed that both pCR and MPR were strongly associated with improved long-term outcomes. pCR versus no pCR and MPR versus no MPR were both associated with better OS and EFS, supporting the prognostic relevance of pathologic response across the available cohorts. These findings support pathologic response as a robust patient-level marker of postoperative prognosis, not merely a morphologic correlate [12].

This association extends into the immunotherapy era. In a meta-analysis of randomized trials of neoadjuvant immune checkpoint blockade, Hines et al. found strong patient-level correlations between pCR or MPR and 2-year EFS. By contrast, the corresponding associations with 2-year OS were weaker and less precise [18].

A practical advantage of MPR over pCR is that it identifies a favorable prognostic subgroup without requiring complete tumor absence, which remains relatively uncommon. In the MD Anderson cohort analyzed by Pataer et al., patients with MPR had significantly better survival than non-MPR patients and showed survival comparable to those with pCR [50]. Thus, MPR identifies a clinically meaningful lower-risk subgroup at the individual-patient level, even if it does not capture the full prognostic information embedded in the resection specimen [17].

### 5.2. RVT as a Continuous Variable

Although the 10% cutoff is operationally useful, recent evidence indicates that RVT behaves biologically and prognostically as a continuous variable rather than a truly binary threshold. The clearest demonstration comes from the prespecified exploratory pathology analysis of CheckMate 816, in which primary-tumor RVT was associated with EFS. In that analysis, 2-year EFS rates were 90% for 0–5% RVT, 60% for >5–30%, 57% for >30–80%, and 39% for >80%, showing progressive outcome worsening with increasing RVT. Each additional 1% increase in RVT was associated with an EFS-HR increase, supporting a graded rather than dichotomous risk model [17].

Nevertheless, moving from a binary to a continuous model does not invalidate MPR. The 10% threshold remains clinically practical, reproducible, and prognostically informative, even if it simplifies a biologic continuum. A recent reproducibility study demonstrated excellent agreement for MPR assessment using the 10% cutoff [29], and the 2025 Cancer Research and Treatment study found that shifting between weighted and unweighted assessment changed MPR status in only one of 81 cases while preserving the 10% threshold as the optimal cutoff for both adenocarcinoma and squamous cell carcinoma in that cohort [38]. Thus, MPR remains useful for reporting, although the underlying prognostic architecture is more accurately understood as continuous [17].

### 5.3. Integrated Primary Tumor and Nodal Assessment

A major refinement in the field has been the recognition that pathologic response assessment cannot be limited to the primary tumor. IASLC recommendations define pCR as the absence of viable tumor in both the primary tumor and sampled lymph nodes, underscoring that nodal findings are integral, rather than ancillary, to treatment-response assessment [3].

Direct evidence for the prognostic relevance of nodal response was provided by Pataer et al., who showed that MPR in regional lymph nodes (LN-MPR) alone significantly predicted long-term OS and that the combination of MPR in the primary tumor (PT-MPR) and LN-MPR stratified patients into three prognostic groups. Patients with PT-MPR had the best outcomes irrespective of LN-MPR status, but among patients without PT-MPR, those who still achieved LN-MPR had a better prognosis than those without response in either compartment [51]. Thus, nodal response adds prognostic information rather than merely mirroring the primary tumor.

Liu et al. extended this concept in a larger cohort by showing that the optimal RVT cutoff in lymph node metastases was 8% and that nodal RVT carried independent prognostic significance, particularly for DFS. The same study found only moderate correlation between primary tumor and nodal response, supporting the notion that these two compartments are biologically related but not redundant [52].

Contemporary neoadjuvant cohorts have further supported the relevance of integrated primary-tumor and nodal assessment, although available studies differ in treatment regimen, endpoint definition, and analytic approach. Huang et al. documented discordant responses between primary tumor and lymph nodes after neoadjuvant chemoimmunotherapy and showed that the combined good-responder group, defined as ypT(pCR+)/ypN0, had the most favorable EFS. Similarly, Cai et al. found that MPR alone was insufficiently discriminative, whereas combining MPR with nodal downstaging improved prognostic performance [53]. These findings support systematic reporting of nodal status and nodal treatment effect, but they do not yet establish a universally accepted composite response score.

Current evidence does not yet provide a validated framework for weighing each nodal station individually within a composite response score. The literature supports the assessment of nodal response and careful examination of involved nodal tissue, but not a definitive hierarchy for single-station versus multi-station residual disease in modern neoadjuvant pathology reporting [51]. Older surgical series suggest that the extent of residual N2 disease matters, particularly on persistent multilevel N2 disease involvement, but these data largely derive from a pre-immunotherapy and often mixed-treatment context [54].

The most defensible conclusion is that nodal response should be systematically incorporated into prognostic interpretation, whereas finer station-by-station modeling remains a future refinement rather than a current standard.

### 5.4. Limits of Surrogacy

The main conceptual limitation of pCR and MPR as surrogate endpoints in neoadjuvant resectable NSCLC is that strong patient-level prognostic value does not, by itself, establish true surrogate validity. Patient-level association refers to whether patients who achieve pCR or MPR experience better outcomes than non-responders. By contrast, trial-level correlation addresses a different question, namely whether treatments that increase pCR or MPR also produce proportional improvements in survival across randomized studies. This distinction is critical: the former supports prognostic relevance, whereas the latter is required to support surrogate validity [55].

Waser et al. addressed this directly. At the patient level, their meta-analysis showed a strong and consistent association between pathologic response and long-term outcomes, with both pCR and MPR associated with substantially improved OS and EFS. However, when the analysis moved from individual patients to treatment effects across trials, the picture changed. At the trial-level, increases in pCR did not consistently translate into proportional improvements in EFS or OS across randomized studies. By contrast, treatment effects on EFS were more closely aligned with treatment effects on OS, suggesting that EFS currently performs better than pCR as a trial-level surrogate of long-term survival [12]. This is consistent with other meta-analytic evidence indicating that improvements in EFS are more likely to translate into improvements in OS after neoadjuvant treatment in resectable NSCLC [56]. Taken together, these findings indicate that pathologic response is clearly prognostic, but that its status as a validated trial-level surrogate remains incomplete.

A similar distinction emerges from the immunotherapy-specific meta-analysis by Hines et al. In that study, pCR and MPR showed strong patient-level relationships with 2-year EFS, indicating that responders had substantially better short-term outcomes than non-responders. However, for pCR and MPR the corresponding associations with 2-year OS were weaker and less precise and trial-level surrogacy for EFS was only moderate and imprecise. The authors therefore concluded that pCR and MPR are promising and clinically informative endpoints, but not yet adequate surrogates for OS in neoadjuvant immunotherapy trials [18].

The prognostic message is strong and consistent. Pathologic response after neoadjuvant therapy identifies patients with more favorable postoperative outcomes [12], and this information becomes even more informative when response is interpreted not only through the binary lens of MPR, but also through the continuous burden of RVT [17] and in conjunction with nodal findings [51,52]. In practical terms, deeper regression generally corresponds to lower risk, and integrated primary tumor plus nodal assessment is more informative than evaluation of the primary tumor alone.

The limits are equally clear. MPR and pCR are robust prognostic markers at the patient level, but they have not yet been fully validated as surrogate endpoints for long-term survival across trials, therapeutic classes, and biologic subgroups [12,18,57]. They should therefore be regarded as highly informative early endpoints and essential components of postoperative risk stratification, but not as substitutes for mature survival data or for integrated clinicopathologic interpretation.

The key missing evidence is therefore not whether responders generally fare better than non-responders, but whether treatment-related increases in pCR, MPR, or reductions in continuous RVT reliably predict proportional improvements in EFS or OS across independent randomized trials. Such validation will require sufficiently mature survival data, harmonized pathology protocols, consistent endpoint definitions, and analyses stratified by treatment class, histologic subtype, and molecular subgroup.

## 6. Moving Beyond a Single Metric: Toward Multidimensional Assessment

The recognition of pathologic response as a composite and multifaceted phenomenon has prompted a conceptual shift toward multidimensional assessment models integrating morphologic, immunologic, and molecular parameters. The objective of these approaches is not to replace RVT assessment, but to determine whether additional features can improve prognostic stratification and provide a more biologically informative representation of treatment response.

### 6.1. Extra Pathologic Variables

Beyond RVT, several additional histopathologic features have emerged as relevant prognostic factors.

Vascular invasion, as well as perineural and pleural invasion, has been associated with worse outcomes even in patients achieving MPR, suggesting that residual invasive potential persists despite apparent tumor regression [44]. Similarly, a high proliferative index in residual tumor, assessed through markers such as Ki67, has been associated with worse OS and may identify biologically active residual disease [44].

Morphometric parameters further refine this evaluation. Quantitative assessment of tumor bed components—including fibrosis and inflammatory infiltrates—has demonstrated prognostic value, particularly when evaluated through objective, reproducible methodologies rather than subjective estimation [44].

The immune microenvironment represents another potentially informative dimension of response. Immune subsets such as CD4+, CD8+, and FOXP3+ lymphocytes, as well as spatially organized immune features, may provide information on antitumor immunity and immunoregulation. However, their prognostic and predictive significance depends on treatment context, tissue compartment, analytic methodology, and spatial distribution, limiting their immediate transferability to routine response assessment [44,58].

**Figure 3:**
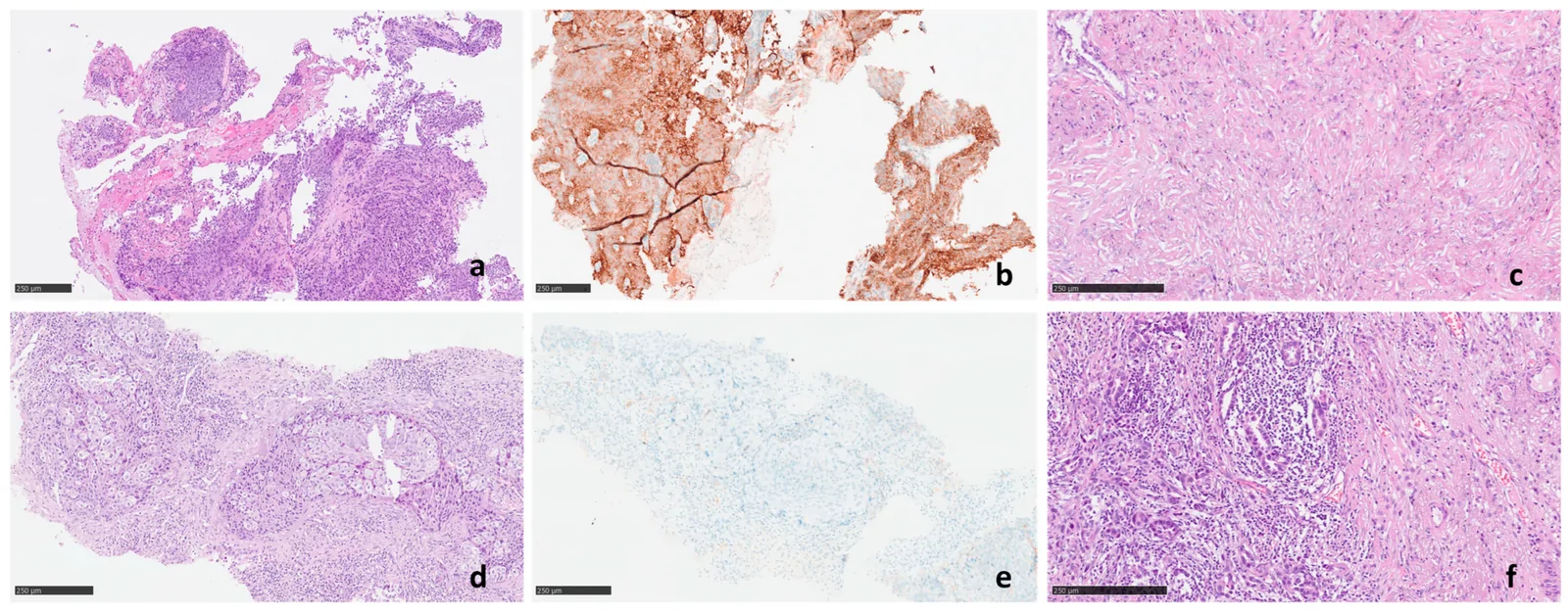
Illustrative examples of PD-L1 expression and pathologic response after neoadjuvant chemoimmunotherapy. Bronchial biopsy showing poorly differentiated NSCLC ((a) hematoxylin and eosin; original magnification 8×) strongly immunoreactive for PD-L1 ((b) immunohistochemistry; original magnification 8×) and corresponding surgical specimen with pCR after neoadjuvant chemoimmunotherapy ((c) hematoxylin and eosin; original magnification 15×). Percutaneous fine-needle biopsy demonstrating adenocarcinoma with predominantly solid pattern ((d) hematoxylin and eosin; original magnification 10×) weakly (TPS 1–49%) immunoreactive for PD-L1 ((e) immunohistochemistry; original magnification 10×) and showing MPR after neoadjuvant chemoimmunotherapy ((f) hematoxylin and eosin; original magnification 15×) on corresponding surgical specimen. These representative cases are intended to illustrate the integration of pretreatment biomarker assessment and post-treatment histopathology, and should not be interpreted, in themselves, as demonstrating a direct predictive relationship between PD-L1 expression and pathologic response. PD-L1 was assessed using the SP263 antibody clone on the Ventana BenchMark platform and scored using the tumor proportion score.

PD-L1 remains the most established tissue biomarker in perioperative immunotherapy studies, but its predictive performance is incomplete and varies across trials and treatment regimens (Figure 3). It should therefore be interpreted as a clinically relevant but imperfect biomarker rather than as a stand-alone predictor of pathologic response. Other molecular or immune variables, including tumor mutational burden and functional T-cell states, remain exploratory and insufficiently standardized for routine response assessment [9,43,58,59,60].

### 6.2. Digital Pathology and Artificial Intelligence

Digital pathology and artificial intelligence (AI) are increasingly being investigated in this field, with the aim of offering tools to operationalize multidimensional assessment.

AI-based models, particularly convolutional neural networks, have demonstrated high concordance with expert pathologists in quantifying RVT and identifying MPR, and may improve the efficiency and scalability of response assessment in research settings [29,61].

Yang et al. addressed a major challenge in the pathologic assessment of NSCLC following neoadjuvant therapy, namely the accurate estimation of MPR in the context of the marked heterogeneity of the tumor bed. The authors developed MMT-Net (Multi-Magnifier Transformer Network), an AI model that combines self-supervised learning, multi-scale feature integration, and transformer-based classification to automatically identify the key components of the tumor bed. Compared with several established AI models, MMT-Net demonstrated superior performance, achieving an accuracy of 99.21% and outperforming models pretrained on natural image datasets, thereby underscoring the value of pathology-specific pretraining. Moreover, attention map analyses revealed that the regions prioritized by the model closely corresponded to those identified by expert pathologists, supporting both the reliability and interpretability of this AI-driven approach for MPR assessment [62]. However, such performance metrics should be interpreted in relation to the unit of analysis, dataset size, validation design, and external validation status. Tile- or region-level classification accuracy, for example, is not automatically equivalent to patient-level MPR classification performance. Moreover, the currently available evidence is still based on isolated studies, often conducted in selected datasets or trial-specific cohorts. Reported performances should therefore be interpreted as proof-of-concept or early validation results rather than as evidence of generalizable routine clinical applicability.

One of the most relevant potential contributions of AI is its role in standardization. By applying consistent algorithms across datasets, AI may help reduce interobserver variability, a known limitation of traditional pathology, particularly in borderline cases or complex tumor beds [38,61,63]. However, implementation outside specialized centers will require validation across scanners, staining protocols, tissue-processing workflows, and institutions, as well as assessment of domain shift, dataset bias, regulatory requirements, interpretability, and integration into routine pathology workflows. Expert pathologist oversight therefore remains essential.

In the LCMC3 study, Dacic et al. evaluated an AI-based approach for the assessment of pathologic response following neoadjuvant atezolizumab in resectable NSCLC. Using whole slide analysis, the algorithm automatically quantified RVT and demonstrated a strong correlation with conventional pathologist-derived assessments [64].

Taken together, these findings support further validation of AI-assisted assessment as a decision-support tool that may complement expert interpretation. At present, however, the evidence remains preliminary and study-specific, and AI should not be regarded as a replacement for standardized histopathologic assessment or expert pathologist oversight.

### 6.3. Targeted Therapy and MRD

The limitations of morphology-based assessment become particularly relevant after targeted therapy, because RVT quantifies local residual tumor burden but does not define the molecular composition, resistant subclones, or systemic relapse potential of residual disease [24,48,49]. This distinction is especially important in oncogene-driven tumors, where a low RVT percentage after tyrosine kinase inhibitor treatment may reflect effective pathway suppression, but conventional MPR and pCR thresholds have not been specifically validated as treatment-agnostic prognostic markers.

This introduces a practical distinction between residual viable tumor and molecular residual disease. RVT is a local histologic estimate within the sampled tumor bed, whereas MRD refers to persistent tumor-derived molecular signals that may be detectable beyond the resection specimen. In localized lung cancer, ctDNA-based assays have shown the ability to identify residual molecular disease and may identify molecular relapse risk before radiologic recurrence [15,16,65]. In the neoadjuvant setting, perioperative ctDNA dynamics have also been explored as biomarkers of treatment response and recurrence risk [66]. However, these observations do not establish ctDNA as a standalone basis for postoperative treatment selection in resectable NSCLC.

ctDNA-based MRD assessment should therefore be regarded as a complementary but still investigational readout. Its interpretation is limited by low tumor shedding in early-stage disease, tumor-informed versus tumor-uninformed assay design, sampling time point, analytical thresholds, and non-uniform definitions of molecular clearance [15,16,65,67]. A negative ctDNA result cannot currently exclude residual disease with certainty, whereas false-positive signals may arise from clonal hematopoiesis rather than tumor-derived DNA [67,68]. Discordance between histologic response and ctDNA status should therefore be interpreted as biologically informative but not yet clinically actionable outside prospective studies.

Histopathology remains the established basis for assessing local morphologic response, including the spatial distribution and characteristics of residual tumor within the tumor bed. ctDNA may provide complementary molecular information beyond the sampled specimen, but its clinical integration after neoadjuvant therapy in resectable NSCLC still requires prospective validation. Future studies should determine whether ctDNA adds reproducible prognostic value beyond standardized RVT assessment, nodal response, and ypTNM stage, and whether combined pathologic–molecular models can be used to stratify patients in prospective escalation or de-escalation trials. Until such validation is available, ctDNA-based management should be considered a hypothesis for prospective clinical trials rather than a routine basis for postoperative treatment modification.

## 7. Future Directions

Pathologic response has become an increasingly important component of treatment evaluation in resectable NSCLC. However, RVT alone cannot fully represent the biological and prognostic complexity of response across different therapeutic classes. Future progress will therefore depend on three closely related priorities: methodological harmonization, prospective validation of integrated response models, and refinement of post-neoadjuvant risk stratification.

### 7.1. Harmonization as the Prerequisite for Progress

The first priority for the field remains methodological harmonization. Although international recommendations have substantially improved consistency in specimen handling and scoring, variability persists across clinical trials and real-world practice in areas such as tumor bed identification, extent of tissue sampling, quantification of RVT, and reporting of nodal response [3]. These differences complicate comparisons among studies and may weaken the reproducibility of pathologic endpoints.

Future trials should therefore adopt standardized pathology protocols, predefined sampling algorithms, uniform endpoint definitions, and centralized or digitally assisted pathology review whenever feasible [3,25,29,64]. Multimodal predictive models may also improve pretreatment stratification, although their incremental value, generalizability, and impact on trial selection require prospective validation [69,70]. For digital pathology and AI-assisted review, broader implementation will also require prospective validation across scanners, staining protocols, tissue-processing workflows, and institutions, together with clear regulatory pathways and integration into routine pathology workflows.

Procedural harmonization is particularly relevant as neoadjuvant strategies expand across different drug classes, where treatment-induced histologic changes may vary substantially. Without robust methodological alignment, even biologically meaningful response markers risk inconsistent interpretation.

In this sense, standardization should not be viewed as a technical afterthought, but rather as the prerequisite for meaningful innovation.

### 7.2. Beyond MPR: Prospective Validation of Composite Response Models

Current pathology endpoints such as MPR and pCR are threshold-based. Although clinically useful, these binary classifications may oversimplify a continuous and biologically heterogeneous process.

Future prospective studies should therefore validate composite response models incorporating multiple parameters, including RVT as a continuous measure, ypN status, lymphovascular invasion, tumor size after therapy, and potentially immune-related histologic features. Such models may outperform isolated MPR or pCR thresholds in predicting recurrence risk and informing trial-based adjuvant treatment strategies.

Demonstrating true surrogate validity will require more than showing that patients with MPR or pCR have better outcomes than non-responders. Future studies should perform formal trial-level surrogate analyses across multiple randomized neoadjuvant trials, testing whether treatment-induced improvements in pCR, MPR, or continuous RVT consistently predict proportional improvements in EFS or OS across treatment arms. These analyses should be prospectively planned whenever possible and stratified by treatment class, histologic subtype, and molecular subgroup. In particular, the conventional 10% RVT cutoff should be further evaluated in adenocarcinoma, squamous cell carcinoma, and oncogene-driven tumors, because its prognostic meaning may not be identical across biologically distinct disease subsets.

Within this framework, ctDNA-based MRD assessment may serve as a future stratification variable rather than an independent therapeutic trigger. Prospective escalation and de-escalation trials should determine whether adding postoperative ctDNA status to standardized RVT assessment, nodal response, and ypTNM stage improves risk prediction and trial-based treatment allocation.

### 7.3. Rethinking Post-Neoadjuvant Staging

Current post-treatment staging relies primarily on ypT and ypN categories, which remain the foundation of prognostic assessment after neoadjuvant therapy. Nevertheless, patients within the same yp stage may have markedly different RVT burdens, degrees of nodal regression, invasive features, and MRD profiles.

Future risk-stratification systems may therefore need to combine residual anatomic disease with depth of treatment response. RVT as a continuous measure, nodal clearance, and validated molecular biomarkers could function as additional prognostic modifiers rather than immediate replacements for ypTNM.

Before incorporation into formal staging systems, these variables must demonstrate reproducibility, independent prognostic value, and clinically meaningful improvement over conventional stage. Their performance should also be validated across histologic subtypes, therapeutic classes, and molecularly defined patient populations.

The most realistic near-term objective is therefore not the replacement of ypTNM, but the development of layered post-neoadjuvant models in which anatomic stage is complemented by standardized pathologic response and, eventually, MRD. Such an approach may better reflect the biological consequences of modern neoadjuvant therapy and provide a more precise framework for postoperative risk assessment.

## 8. Conclusions

Pathologic response assessment in resectable NSCLC has evolved from a descriptive post-treatment observation into a standardized endpoint with established patient-level prognostic value and an increasingly central role in neoadjuvant clinical trials. pCR and MPR identify patients with favorable outcomes, although their validity as trial-level surrogates for long-term survival remains incompletely established, particularly across different therapeutic classes.

The IASLC recommendations provide the essential methodological foundation for reproducible assessment through standardized tumor bed identification, tissue sampling, RVT quantification, and nodal evaluation. Within this framework, MPR remains a practical reporting threshold, but RVT is more accurately understood as a continuous prognostic variable, and its interpretation may be strengthened by integration with nodal response and other validated clinico-pathologic features.

Immune and stromal characteristics, digital pathology, AI, and ctDNA-based MRD assessment may further refine treatment-response evaluation and postoperative risk stratification. However, these approaches should currently be regarded as complementary and investigational rather than replacements for standardized histopathology.

Future progress will depend on prospective multicenter validation of integrated models capable of demonstrating reproducibility and prognostic value beyond MPR, pCR, and ypTNM staging.

## Figures and Tables

**Table 1:** Pathologic response endpoints after neoadjuvant therapy in resectable NSCLC.

Endpoint	Assessed Compartment	Definition or Reporting Approach	Current Level of Standardization	Main Use and Evidence
**Primary-** **tumor RVT**	Primary tumor bed	Percentage of residual viable tumor within the primary tumor bed, assessed together with necrosis and stroma so that the three components sum to 100%	Standardized in IASLC recommendations	Core quantitative measure for pathologic response assessment; increasingly supported as a continuous prognostic variable
**Primary-tumor MPR/PT-MPR**	Primary tumor bed	Conventionally defined as 10% or less residual viable tumor in the primary tumor bed	Widely adopted and standardized as the operational definition of MPR in NSCLC	Practical binary endpoint for reporting and clinical trials; robust patient-level prognostic marker
**pCR**	Primary tumor and sampled regional lymph nodes	Complete absence of viable tumor in both the resected primary tumor and sampled regional lymph nodes	Standardized and widely used	Strong favorable prognostic marker at the patient level and major endpoint in neoadjuvant trials
**Nodal** **treatment** **response/nodal RVT**	Regional lymph nodes	Presence or absence of residual nodal metastasis and, when residual metastatic tissue is present, descriptive assessment of treatment effect and viable tumor burden	Recognized as clinically relevant but not incorporated into a universally standardized IASLC-endorsed quantitative response score	Provides prognostic information complementary to primary-tumor response and should be clearly communicated in multidisciplinary discussion
**LN-MPR**	Regional lymph node metastases	Low residual viable tumor burden in nodal metastases; proposed thresholds have varied across studies	Investigational; no universally accepted cutoff	Reported in selected retrospective studies as prognostically informative but should not be treated as equivalent to primary-tumor MPR in routine reporting
**ypN0/nodal clearance**	Regional lymph nodes	Absence of residual regional lymph node metastasis after neoadjuvant therapy and surgery	Standardized within ypTNM staging	Established component of post-treatment staging; complements primary-tumor response in postoperative risk stratification
**Composite primary-** **tumor plus nodal** **response**	Primary tumor and regional lymph nodes	Integrated models combining primary-tumor RVT or MPR with nodal response, ypN status, or nodal downstaging	Emerging and not yet standardized	May improve prognostic stratification beyond primary-tumor response alone, but requires prospective validation before routine use as a formal response endpoint
**ypTNM stage**	Primary tumor, regional lymph nodes, and metastasis categories	Post-treatment anatomic stage assigned after neoadjuvant therapy and surgery	Standardized staging framework	Remains the foundation of postoperative prognostic assessment; pathologic response variables should currently complement rather than replace ypTNM

**Table 2:** Therapy-related regression patterns and interpretative caveats in neoadjuvant-treated NSCLC.

Treatment Setting	Commonly Described Regression Features	Main Interpretative Caveats	Current Status
**Chemotherapy**	Necrosis, hypocellularity, stromal fibrosis, fibroblast proliferation, and heterogeneous inflammatory infiltrates. Residual viable tumor may persist as irregular or discontinuous foci within fibrotic or necrotic areas.	Necrosis and fibrosis are not specific indicators of treatment effect, because they may also reflect pretreatment tumor biology or spontaneous regression-like change. Limited sampling may underestimate RVT in heterogeneous tumor beds.	Established historical setting for RVT-based response assessment and development of MPR as a practical endpoint.
**ICI monotherapy**	Immune-rich fibroinflammatory regression bed with lymphocytes, histiocytes, foamy macrophages, cholesterol clefts, granulomatous reaction, proliferative fibrosis, neovascularization, and tertiary lymphoid structures. An outside-in pattern of regression has been described.	Immune-related features are biologically informative but not individually specific. They should complement, not replace, direct quantification of viable tumor. Evidence derives largely from selected neoadjuvant anti-PD-1studies.	Immune-related regression criteria are useful descriptive tools, but standardized response assessment remains based on RVT, MPR, and pCR.
**Chemo-** **immunotherapy**	Overlapping cytotoxic and immune-mediated changes, including tumor-cell death, fibrosis, necrosis, macrophage-rich clearance, lymphoid aggregates, and tissue repair within the same tumor bed.	Individual morphologic findings cannot usually be attributed exclusively to chemotherapy or immune checkpoint blockade. Treatment-related patterns may overlap, and regimen-specific criteria are not validated.	Most relevant contemporary clinical-trial setting; pathologic response endpoints are widely used, but qualitative regression features remain complementary descriptors.
**Targeted therapy/TKIs**	Reduced tumor cellularity, variable fibrosis and necrosis, generally less prominent immune activation than in ICI-treated tumors, and persistent foci of viable carcinoma in a remodeled tumor bed.	Available data are limited and heterogeneous across driver alterations, agents, treatment duration, sampling, and response definitions. Morphology alone cannot identify resistant subclones, and MPR/pCR thresholds require dedicated validation in this setting.	Emerging setting; no reproducible therapy- specific histologic criteria or validated targeted therapy-specific response thresholds are currently established.

## Data Availability

No new data were created or analyzed in this study. Data sharing is not applicable to this article.
